# Health literacy in physical trauma patients: Evaluation of the general understanding of patients in an outpatient clinic using the German Short Test of Functional Health Literacy

**DOI:** 10.1007/s00508-024-02389-3

**Published:** 2024-07-31

**Authors:** Alexandra Christ, Oskar Bamer, Jesse Seilern und Aspang, Silke Aldrian, Johannes Herold, Thomas Haider

**Affiliations:** 1 Department of Plastic, Reconstructive and Aesthetic Surgery, Medical University of Vienna, Vienna, Austria; 2 Department of Orthopedics and Trauma Surgery, Medical University of Vienna, Vienna, Austria

**Keywords:** German S-TOFHLA, Validation, Physical trauma patients, Outpatient facilty, Medical information comprehension, Sociodemographic factors, Patient education

## Abstract

**Background:**

Health literacy (HL) refers to the ability to understand and process information provided by the healthcare system and depends on various factors, such as language comprehension, education, and social environment. Low HL was recently associated with increased readmission, morbidity, and mortality. Little is known about HL levels in physical trauma patients. The aim of this study was to determine general HL in physical trauma patients in an outpatient setting and to evaluate possible differences based on demographic characteristics.

**Material and methods:**

A total of 100 physical trauma patients were recruited in the outpatient trauma facility of the Medical University of Vienna. All recruited patients completed the German Short Test of Functional Health Literacy (S-TOFHLA).

**Results:**

The evaluated HL index ranged between 20 and 36 points (highest achievable score: 36 points), with the mean value calculated at 34.3 (adequate). Out of 100 participants, 97 patients (97%) showed adequate HL and 3 patients (3%) reached a score corresponding to a marginal understanding. No patient showed inadequate HL utilizing the S‑TOFHLA tool. No significant differences were found between different demographic categories, including age, education level, native language, and injury location.

**Conclusion:**

In this study, included outpatient trauma patients demonstrated an overall adequate understanding of healthcare related information. Age, sociodemographic background, and/or educational status did not influence performance, which leads to the question as to whether the German version of the S‑TOFHLA is valid to representatively measure HL in these patients. Furthermore, regarding the obvious shortcomings of the S‑TOFHLA, the education standard of the respective population should be taken into consideration when choosing an appropriate testing tool.

## Background

Health literacy (HL) is defined as the understanding of healthcare information, its adherence and application, the understanding of the need for follow-up care or regular medication regimens, and its execution [1, 2]. Different levels of HL are significantly connected to the performance of preventive examinations, increased hospitalization, outcome of diseases, and influence regarding the healthcare system [3, 4]. Moreover, low HL can have major financial repercussions on the healthcare system itself, caused by the patient’s inability to properly assess potential risk factors, especially in follow-up care [3]. In addition to the increased overall costs connected to higher healthcare utilization, it can influence adherence to health-related interventions, mortality, and readmissions [4].

Various studies, mostly conducted in the USA, showed poor to marginal HL among 25–46% of the population [5–7]. These authors used different methods to assess HL, such as the short assessment of health literacy (SAHL) and a version of the short form-interpersonal processes of care (SF-IPC) or the English version of the Test of Functional Health Literacy (TOFHLA) and its shorter version, the Short Test of Functional Health Literacy (S‑TOFHLA) questionnaires [8].

The topic of HL has been discussed throughout several studies in the past, including patients who suffered from heart failure and multimorbid cardiac patients as well as increased translational care needs in hospitalized patients and families with children, focusing on the parent’s impact [9–12]. All of these studies strongly promote the necessity of adequate HL among patients due to its influence on both the patient’s well-being and the healthcare system.

Levels of understanding healthcare information vary between individuals and depend on various factors, such as spoken language, social environment, and educational status, as well as different professions [1]. Poor HL also seems to be highly connected to higher age, lower income, and higher number of comorbidities [9, 11, 12].

In 2015, Rosenbaum et al. used the developed and validated literacy in musculoskeletal problems (LiMP) questionnaire to show that 33% of Americans have inadequate musculoskeletal HL; however, significant differences can be detected between ethnic backgrounds and levels of education [3]. Compared to the USA, patients visiting the trauma outpatient department in Europe mostly present with blunt trauma caused by events such as falls, which is why there is a strong correlation between musculoskeletal HL and trauma HL in this patient population [13].

There are currently few studies, e.g., Rosenbaum et al. (2015), that address outpatient physical trauma patients and musculoskeletal HL. This study assessed the patient’s general language understanding as well as a HL which can be related to the understanding of physical trauma-related injuries and the consequences. It is important to note that the German version of the S‑TOFHLA was used to evaluate the general HL in patients at this outpatient clinic rather than musculoskeletal HL.

To our knowledge, this is the first study to report health literacy in outpatient physical trauma patients in Austria.

## Material and methods

The question was posed whether an adequate understanding of health topics is compromised by patient background or age. We further assessed potential correlations between sociodemographic parameters and overall HL scores, using a validated tool. As only descriptive data analysis was conducted, no specific hypothesis has been established.

Data collection: 100 physical trauma patients were examined at the local outpatient trauma facility of the Medical University of Vienna and agreed to participate in the HL section of the German S-TOFHLA. This is a test published by Baker et al. in 1999, modified from the original TOFHLA and shortened to 36 items [8]. The anonymized questionnaire was distributed among the patients at various random time points in the emergency waiting room. All patients present in the waiting room were asked whether they would be interested in taking part in an anonymized questionnaire study.

The doctors responsible did not make any preliminary decisions about which patients they would like to include in the study or not.

Preliminary information about the evaluation of the test and its intent was given to everyone interested in participating. Patients not willing to and/or not capable (due to health or cognitive reasons, not due to language barrier) of completing the questionnaire were excluded from participating in the study. All patients were included until 100 participants completed the S‑TOFHLA. Ethical approval was not needed as it is only the anonymized data from the questionnaire without being linked to medical data.

Demographic information, including gender, age, profession, highest form of education, and type of injury, was recorded.

S‑TOFHLA evaluation: the S‑TOFHLA questionnaire involves 36 single-choice questions, giving 4 possible answers with a single correct answer. The score is divided into the following subgroups: 36–23 adequate, 22–17 marginal, 16 and less inadequate [8, 14].

Statistical analysis: descriptive categorical data are displayed in relative numbers and percentages, which are represented by the same number due to the total number of participants being exactly 100. SPSS Statistics 27.0.1 (IBM Corp., 2020, Armonk, NY, USA) was used for statistical analysis. The Mann-Whitney U test was used to compare S‑TOFHLA scores across categories with two levels (gender, German/not German as the mother tongue, and single/multiple injuries) and the Kruskal-Wallis test for those with more than two levels (education, occupational status, age group and localization of current injuries). Cases in certain categories were excluded from comparison if the sample size was too small.

Due to variable sample sizes and number of groups regarding the testing of each sociodemographic parameter according to an empirical population observation in a trauma outpatient clinic, estimated effect sizes were calculated using G*Power 3.1 (Faul, Erdfelder, Lang, Buchner, 2007). When conducting comparisons with the Mann-Whitney U test, the estimated effect size is calculated as d = 0.75–1.28, depending on the group’s sample size. Applying the Kruskal-Wallis test, the estimated effect size is calculated as f = 0.4–0.42, depending on the number of compared groups.

The alpha level was set at 0.05 for each comparison to determine statistical significance.

Examined categories as mentioned above, such as education, occupational status, age, and native language were selected based on the current literature results, where strong associations and significant differences have been reported in general HL understanding.

Furthermore, hardly any direct comparisons between patient HL levels throughout different departments have been reported, which could promote department-specific individual HL communication. Pursuing this idea on a smaller scale, we additionally included trauma-specific subdivisions regarding respective injury locations and the number of injuries (single/multiple).

## Results

The cohort included 51 female and 49 male patients with an average age of 36.7 years (range 18–87 years; standard deviation 13.94 years). Of the patients 91 declared a German-speaking country as their place of birth, and the remaining 9 stated a mother tongue other than German. Descriptive statistical data regarding the categorization of the subjects, as well as general data, are displayed in Table 1.

**Table 1:** Baseline characteristics of participants included in the study

	*n* *=* *100*
*Gender, %*	
Male	49
Female	51
*Age in years, mean (SD)*	36.72 (13.94)
*Education Status, %*	
No high school diploma	13
High school diploma	38
Higher education	49
*Occupation status, %*	
Unemployed	5
Employed	73
Retired	6
Student	16
*German-speaking country of birth, %*	
Yes	91
No	9
*Localization of main injury, %*	
Head	8
Thorax and spine	5
Upper limbs	40
Lower limbs	42
Upper and lower limbs	5
*Single or multiple injuries, %*	
Single	90
Multiple	10

*SD* standard deviation

Overall, we found a mean score of 34.3 (±2.8), 97 patients demonstrated an adequate understanding of health topics with a mean score of 35.7 (±1.433). The remaining 3 patients ranked in the marginal range, with a mean score of 20.6 (±0.58). No participant showed an inadequate HL score.

A slight tendency towards a negative correlation between age and HL did not reach significance (Spearman correlation test, r = −0.157, *p* = 0.12).

**Fig. 1:**
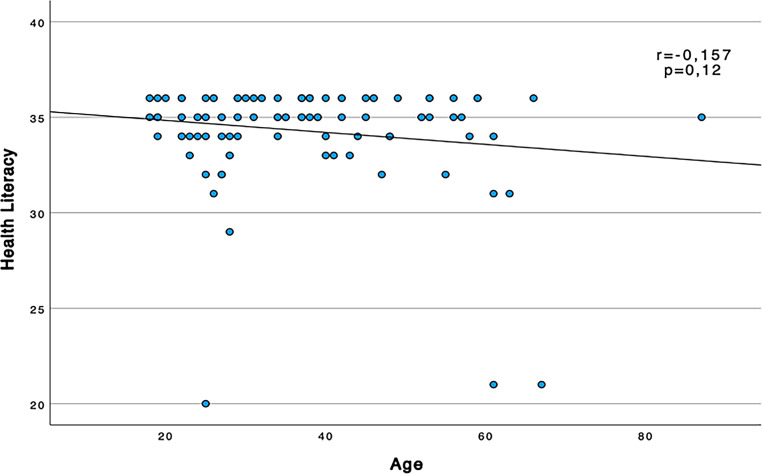
Spearman’s rank correlation between health literacy score (points using the S‑TOFHLA tool) and age of patients (years)

The corresponding scatter plot is shown in Fig. 1.

Comparing the evaluated demographic parameters, we did not find any statistically significant differences between the groups. These comparisons are shown in Table 2.

**Table 2:** Health literacy index (S-TOFHLA) by patients’ characteristics (*p*-values from Mann-Whitney and Kruskal-Wallis tests)

*n* *=* *100*	Mean value (minimum–maximum) [SD]	*p*-value
*Gender*		0.957^a^
Male	34.47 (21–36) [2.36]	–
Female	34.16 (20–36) [3.17]	–
*Age in years, categorized*		0.547^b^
≤ 25 (*n* = 25)	34.24 (20–36) [3.14]	–
26–35 (*n* = 28)	34.46 (29–36) [1.75]	–
36–50 (*n* = 28)	35 (32–36) [1.19]	–
50 < (*n* = 19)	33.16 (21–36) [4.57]	–
*Education Status*		0.26^b^
No high school diploma	32.38 (21–36) [5.22]	–
High school diploma	34.32 (20–36) [2.89]	–
Higher education	34.82 (31–36) [1.27]	–
*Occupation status (n* *=* *89)*		0.738^a^
Unemployed^c^	33.8 (31–36) [2.59]	–
Employed	34.59 (20–36) [2.16]	–
Retired^c^	30 (21–36) [7.21]	–
Student	34.81 (32–36) [1.38]	–
*German-speaking country of birth*		0.172^a^
Yes	34.46 (21–36) [2.48]	–
No	32.78 (20–36) [4.97]	–
*Localization of injury (n* *=* *82)*		0.790^a^
Head^c^	32.88 (20–36) [5.46]	–
Thorax and spine^c^	33.8 (29–36) [2.78]	–
Upper limbs	34.08 (21–36) [3.32]	–
Lower limbs	34.79 (31–36) [1.3]	–
Upper and lower limbs^c^	35 (34–36) [1]	–
*Single or multiple injuries*		0.742^a^
Single	34.28 (20–36) [2.92]	–
Multiple	34.6 (32–36) [1.35]	–

*S‑TOFHLA* short test of functional health literacy, *SD* standard deviation

^a^Mann-Whitney U test

^b^Kruskal-Wallis test

^c^Excluded from statistical comparison due to insufficient sample size

While individuals with a primary school education as their highest level of education score had an average of 32.38 points, participants with a high school education average 34.32 points, which corresponds to a mean difference to the lower level of education of 6% (1.94 points). For patients with a college degree, the average score was 34.82 points, representing a 7.5% (2.44 points) difference to the lowest level of education and a 1.4% (0.5 points) difference to the middle level of education.

Furthermore, 2 out of 3 patients in the marginal range of S‑TOFHLA are represented in the group with the lowest educational attainment (primary school), both also being over 60 years old. The third low scorer, while not being born in a German-speaking country, was located in the age group (≤ 25 years) with a high school diploma as highest education level.

Participants with a mother tongue other than German, or differences in location and number of injuries did not significantly differ regarding their HL.

### Ex post calculations

As for the validity of the German S‑TOFHLA, this study may have proven certain limitations and weaknesses regarding the test evaluation subgrouping. As indicated in the results 97% showed an overall adequate HL with a mean result of 34.72 (1.43 SD) out of 36 points, leading to a high ceiling effect and potentially concealing significant differences.

To further pursue this theory a single new cut-off score at 32 points was selected, dividing the final HL score into either adequate or inadequate.

We decided to dichotomize and re-evalute certain sociodemographic categories, dividing the educational aspect into high school degree and higher forms of degree (*n* = 87) or the absence of a high school degree (*n* = 13). In addition, the participant’s age was adjusted into 2 groups, using 60 years as a cut-off parameter (below 60 years: *n* = 93; above 60 years: *n* = 7).

To check whether adequate and inadequate S‑TOFHLA scores were distributed equally amongst age and educational groups, Fisher’s exact test (= exact χ^2^-test) was applied. Significant associations (*p* = 0.05) between adequate and inadequate S‑TOFHLA scores and the re-evaluated groups were determined. Therefore, the null hypothesis can be rejected in both cases.

The modified analysis revealed compelling evidence of associations between educational status and HL, with a significance level of *p* = 0.048. Furthermore, a highly significant association (*p* = 0.004) was found between individuals aged over 60 years and HL. Other demographic parameters, including speaking German as first language, did not reach significance.

## Discussion

In comparison to the 25–46% [5–7] of overall patients with low or marginal HL mentioned in previous studies, patients in this outpatient setting achieved far better results. It can be argued that patients who explicitly visit the trauma outpatient department have a certain level of health understanding. This could be explained by the distinct separation of the trauma department and early triage in German-speaking countries of physical trauma patients from other general emergency patients.

The 100 randomly selected patients showed considerable diversity in age and educational level. Based on the data of this study, there is no statistical significance in the correlation between HL and age. Nevertheless, as shown in the figure (Fig. 1), we did observe a tendency for HL to decrease with age, which has already been described in the literature [9]. Due to the sociodemographic trends of an aging society, this may indicate that in the future an increasing number of older people with low HL will emerge. Although not statistically significant, lower HL was found in patients with lower levels of education. This has also been shown in several previous studies [15].

Moreover, no significant difference in HL between patients born in German-speaking countries and patients with another mother tongue was apparent in this cohort.

Following the results of this study with the German S‑TOFHLA, there is no reason to believe that HL in trauma patients is severely impaired; however, due to the separation of injuries and emergencies in the Austrian triage system, no information can be provided regarding other emergencies. Furthermore, the instrument used shows ceiling effects, so that results have to be interpreted carefully.

The educational data from Statistics Austria showed that in 2020, 50.5% of the Austrian population had no high school diploma, 30.4% had a high school diploma and 19.1% had a higher educational qualification. Compared to our patient population, there were many more patients with a higher level of education, which could also explain the ceiling effect [16].

Although the German version of the S‑TOFHLA has been validated, the question still arises as to whether the questionnaire itself or the scoring system should be re-evaluated [14].

The S‑TOFHLA in this sample had a marked ceiling effect, which reduces or even removes its ability to discriminate between groups that may indeed differ in HL. An indication of this failure of S‑TOFHLA was found by re-evaluating the findings after dichotomizing the score at a value of 32, which resulted in significant differences for educational and age groups.

We additionally assessed the width of the S‑TOFHLA subgroups as too broad and therefore lacking informative value. By narrowing down the threshold and including the former “marginal” evaluation within the “inadequate” group, we have been able to attain significant differences; however, we are convinced that in larger study groups significant results could be possible even with 3 subgroups (adequate, marginal, and inadequate). Alternatively, the patient collective with a similar distribution of the level of education as in the data from Statistics Austria would be more conclusive. While an overall outstanding HL apprehension of outpatient physical trauma patients is a desirable outcome welcomed by medical staff, overestimation of general understanding could lead to potential risks.

In contrast to these findings, a systematic review of studies applying the S‑TOFHLA in the USA found that 10.5–32% of patients showed marginal or inadequate HL [7].

Interestingly, in Connor et al.’s (2013) Swiss validation study on the German S‑TOFHLA version, a total of 233 participants (93.6%) showed adequate HL. Only 7 participants (2.8%) showed a marginal score, and 9 (3.6%) presented an inadequate score. These results, although not acquired in an emergency department setting, are in good agreement with our findings [14].

This could be interpreted as an overall higher score using the German S‑TOFHLA. Whether those test results are generated due to an easier test version or indeed due to higher skills, shown e.g., in reading comprehension or HL, is yet to be determined and would require more follow-up studies.

Although attempts to validate the German version of the S‑TOFHLA have been undertaken [14], we still advocate revising this instrument due to its inefficient discriminatory power.

Concerning our initial results following the validated HL questionnaire scoring system 2 of the 3 patients considered with a marginal result were older than 60 years, further contributing to the discussion of whether the current evaluation displays a reliable measurement tool. The third low scorer came from a non-German speaking country and although of higher education was not sufficiently fluent in German to score high in the S‑TOFHLA. Hence, the overall poor HL could be significantly higher than that evaluated; however, it is unclear to what extent the data collected in this study are compromised by this language issue.

Providing an additional validated section, where potential poor results can be further attributed to either language or understanding could supply valuable insights and help improve the delivery of medical information.

Facing patients with an overall inadequate understanding, simplification of complex medical terminology as well as avoidance of technical terms should be thoroughly practiced.

Moreover, incorporation of visual aids such as diagrams, charts, and illustrations can be utilized to supplement written or spoken information. Through this visual assistance, complex concepts can be conveyed more effectively for individuals with limited literacy skills or language proficiency [17].

When engaging in a conversation the provider of medical information should ensure and promote two-way communication to ensure comprehension and address any questions or concerns patients may have. Compliant patients can be frequently asked to repeat or summarize certain aspects throughout the conversation. Especially with multilayered, codependent, and more complex information, this principle can ensure the comprehension of each step [17].

A cultural component is also to be considered regarding communication with patients. It is crucial to recognize cultural differences in communication styles, beliefs, and practices when delivering healthcare information and to tailor communication approaches to resonate with diverse cultural backgrounds [18].

If the insufficient understanding is due to a pronounced language barrier, brochures and pamphlets should always be provided in multiple languages to accommodate individuals who may not be fluent in the local language [19]. Additionally, interpretation services such as in-house interpreters or online language tools for real-time communication should be available to avoid misunderstandings [20, 21].

Due to the ceiling effect, which was already shown in the German-speaking collective of Connor et al. (2013) and is reproduced in this study, the question was raised, whether the German S‑TOFHLA is accurately validated [14]. This could mean that the German S‑TOFHLA needs a modification, either in its cut-off values or in the translation of the questions from the original English S‑TOFHLA. Based on the study design this statement can currently be applied to outpatient physical trauma patients only; however, it must also be taken into consideration that the concept of HL looks different in various parts of the world. Before implementing and using tools such as the S‑TOFHLA, the requirements for HL itself as well as the resulting adequate communication between healthcare provider and patient should ideally be analyzed.

While Özdemir et al. (2020) considered their study population in the emergency department to have an overall lower HL than the average population [1], we cannot support this assumption based on our results or missing reference values from other local medical departments. We found a significantly lower percentage of patients with marginal or inadequate HL, potentially caused by the instrument used. Even after the re-evaluation of our results using the modified scoring system, we observed a greater overall understanding of medical information in our study.

While the consequences of low HL may be limited in the outpatient setting, its impact may be of a much greater scale in the inpatient setting caused by the greater amount of medical healthcare information and the requirement for better understanding, compliance, and following more complex instructions. In this respect, there are few to no studies regarding physical trauma patients.

The questionnaire study can only provide a momentary perspective at the time of the outpatient presentation. The extent to which patients now follow the medical instructions and the posttreatment regimen cannot be determined on the basis of the evaluations of this study. To obtain the best possible results, a prospective study could be conducted in which not only questionnaires such as the S‑TOFHLA are completed by patients but also correlations are made with compliance in the posttreatment regimen. This could provide more concrete information about HL in outpatient physical trauma patients.

### Limitations

Overall, the system-based sorting of physical trauma patients could impose a certain bias, as a basic level of HL can be expected if a patient knows which department to visit based on their medical condition. Another limitation is the fact that only data from complete questionnaires were analyzed. By handing the questionnaires out personally, the attending resident may have inflicted a personal bias on the composition of the study group, based on the impression, of whether a patient would complete the S‑TOFHLA.

## Conclusion

This study found that outpatient physical trauma patients showed overall good results in HL when using the aforementioned re-evaluated scoring system. When using cut-off values suggested by S‑TOFHLA no significant differences were observed regarding age, socioeconomic background, and/or educational status. Selecting higher cut-off values as well as a narrower subgrouping we did find a significant association between higher age and lower educational status with inadequate HL. Whether this outcome can be explained by health system-related better HL or is the result of an easier test version of the German S‑TOFHLA is yet to be determined. Based on our findings following the German S‑TOFHLA evaluation in reference to a high ceiling effect as well as too broad subgrouping, we advocate a revised version and doubt its current validity. Moreover, the issue of language barriers leading to a poor HL score cannot be adequately addressed in a standardized test such as the S‑TOFHLA. Potential ways to incorporate this topic in prospective HL testing methods should be further explored. As of now, the lack of questionnaires with validated and reliable translations in multiple languages is a subject to be further addressed. Performing future studies using validated context-specific assessment tools could provide more accurate data on HL in physical trauma patients.

## Data Availability

The datasets generated during and/or analyzed during the current study are available from the corresponding author upon reasonable request.

## Abbreviations

HL: Health literacy
S‑TOFHLA: Short test of functional health literacy
TOFHLA: Test of functional health literacy
SD: Standard deviation
